# Systematic review of feeding difficulties in children with eosinophilic esophagitis: An EAACI Task Force report

**DOI:** 10.1111/pai.70087

**Published:** 2025-04-17

**Authors:** Maria Ruano‐Zaragoza, Sarah‐Anne Hill, Ulugbek Nurmatov, Imke Reese, Mario C. Vieira, Christophe Dupont, Carina Venter, Joanne Walsh, Glauce Yonamine, Alexia Beauregard, Fernanda González‐Matamala, Antonella Cianferoni, Audrey DunnGalvin, Marta Vazquez‐Ortiz, Rosan Meyer

**Affiliations:** ^1^ Allergy Department Hospital Clínic Barcelona Barcelona Spain; ^2^ Clinical & Experimental Respiratory Immunoallergy Institut Investigacions Biomediques August Pi I Sunyer (IDIBAPS) Barcelona Spain; ^3^ National Heart and Lung Institute Imperial College London London UK; ^4^ Division of Population Medicine, School of Medicine Cardiff University Cardiff UK; ^5^ Nutrition Therapy Munich Germany; ^6^ Center for Pediatric Gastroenterology Hospital Pequeño Principe Curitiba Brazil; ^7^ Paris Descartes University Paris France; ^8^ Ramsay Group Clinique Marcel Sembat Boulogne Billancourt France; ^9^ University of Colorado/Children's Hospital Colorado Denver Colorado USA; ^10^ Castle Partnership NHS Norwich UK; ^11^ Division of Nutrition, Instituto da Criança e do Adolescente Hospital das Clínicas da Universidade de São Paulo São Paulo Brazil; ^12^ Faculty, Ellyn Satter Institute, Clinical Dietetics Branch Winn Army Community Hospital Fort Stewart Georgia USA; ^13^ Allergy and Immunology Division, Perelman School of Medicine, The Children's Hospital of Philadelphia University of Pennsylvania Philadelphia Pennsylvania USA; ^14^ NIHR Southampton Respiratory Biomedical Research Unit, University Hospital Southampton NHS Foundation Trust University of Southampton, Faculty of Medicine Southampton UK; ^15^ Imperial College London London UK; ^16^ University of Winchester Winchester UK; ^17^ University of KU Leuven Leuven Belgium

**Keywords:** eating difficulties, eosinophilic esophagitis, feeding difficulties, prevalence, systematic review

## Abstract

The term “feeding difficulties” (FD) encompasses a range of phenotypes characterized by inadequate food intake and/or inappropriate eating habits for a given age. Eosinophilic esophagitis (EoE) is a chronic, immune‐mediated condition often affecting children. It leads to esophageal dysmotility, potentially impacting feeding/eating. However, little is known regarding the true prevalence of feeding/eating difficulties in children with EoE. The main objective of this systematic review was to address this knowledge gap and determine the impact of FD in children with EoE. We searched eight international databases for all published studies from inception until March 2024. All publications were screened against pre‐defined eligibility criteria and critically appraised by established instruments. The substantial heterogeneity of included studies precluded meta‐analyses, so a narrative synthesis of quantitative data was performed. A total of 3442 abstracts were assessed, 29 underwent full‐text screening. Ten studies met eligibility criteria and were analyzed. Across these, 18 different terms to define FD and 6 diagnostic tools were used. All included papers reported quantitative data on the FD prevalence in children with EoE, ranging from 13% to 75.3%. Concomitant IgE food sensitization/allergy was common (26.2%–88%) but its impact on FD occurrence was unclear. The current literature suggests that FD is prevalent among children with EoE, particularly those with associated IgE‐mediated food allergies. However, the heterogeneity of terminologies and diagnostic tools makes drawing conclusions challenging, as it might have impacted outcomes. Further research and guidance on the diagnosis and management of FD in children with EoE are needed to appropriately identify and manage such patients.

**Key message**

Feeding difficulties are prevalent in children with eosinophilic esophagitis, particularly those with concomitant IgE‐mediated food sensitization. There is currently no consensus on how to assess feeding difficulties in children with eosinophilic esophagitis, and great heterogeneity of definitions and diagnostic criteria has been found across the literature. Future work should focus on developing such tools to harmonize the formulation of diagnostic and treatment guidelines and therefore improve clinical outcomes, as well as aid prospective research in this field.

## 1 INTRODUCTION

Feeding difficulties (FD) encompass a spectrum of phenotypes, characterized by suboptimal food intake and/or lack of age‐appropriate eating habits.^1^ Various feeding difficulty terminologies are used in literature and clinical practice, across pediatric populations, including food allergies,^2^ with a glossary of terminologies provided in Table 1. Presentations may include disruptive mealtime behavior, food selectivity, or aversions due to discomfort, pain, or traumatic events like food impaction or allergic reactions.^3^, ^4^, ^5^, ^6^

**TABLE 1:** Glossary of terms for the feeding difficulties included in this systematic review.

Feeding difficulty	Definition
Aversive/avoidant eating	Strategies of eating resulting from repeated experiences of physical or emotional pain or discomfort during feedings, to avoid the aversive feeding situations
Behavioral feeding difficulty	Broad term used to describe a variety of problematic mealtime behaviors including, among others: throwing food, refusal to sit at a table and screaming to avoid the meal
Eating too little/no appetite	Lack of hunger resulting in eating too few calories for age/size/reliance on enteral feeding for appropriate calorie intake
Fear of food	Irrational fear of eating that prevents enjoyment of food and affects daily life; it can be specific to one type of food or many
Feeding difficulties/problems/dysfunction	Generic terms, characterized by suboptimal intake of food and/or lack of age‐appropriate eating habits (includes all feeding difficulty phenotypes)
Food aversion	Refusal of foods that are presented to the child despite being developmentally appropriate
Food refusal	Refusal by individual to eat all/most foods presented to them; failure to ingest adequate nutrition to maintain appropriate weight for age/size
Fussy eating	Often used interchangeably with picky eating. Inadequate variety/quantity of foods through rejection of both familiar and unfamiliar foods, often in an inconsistent pattern
Maladaptive feeding	Caregiver use of inappropriate strategies to improve the child's nutritional status, which perpetuate/worsen malnutrition and other manifestations of feeding dysfunction
Pediatric feeding disorder	Impaired oral intake that is not age‐ appropriate, and is associated with medical, nutritional, feeding skill, and/or psychosocial dysfunction
Picky eating	Often used interchangeably with fussy eating. Eating a limited variety of foods/unwilling to try new foods, despite the ability to eat a broader diet, as well as strong food preferences
Selective eating	Strict rules on the color, texture, taste and the way the food is cooked
Slow eater	Mealtime duration >30 min

FD ranges from mild to severe, with severe cases, including Avoidant Restrictive Food Intake Disorder (ARFID), impacting growth, cognitive function, social interactions, caregiver mental health, and quality of life (QoL).^7^, ^8^, ^9^, ^10^, ^11^ Feeding/Eating disorders like ARFID may have long‐term adverse effects on both health and psychological well‐being.^11^, ^12^, ^13^, ^14^

It is well reported in the literature that a significant proportion of children experience periods of food refusal as they become more autonomous, and food neophobia is seen as part of the development of all omnivores.^14^, ^15^, ^16^

FD affect 25%–45% of the general pediatric population, 80% of children with developmental disabilities, and 40%–70% with chronic conditions (such as disorders that affect oral, nasal, or pharyngeal function, aerodigestive disease or neurologic, developmental, and psychiatric disorders),^11^ while in IgE and non‐IgE mediated food allergy, prevalence ranges from 13.6% to 40%.^2^

Children with EoE face unique challenges contributing to FD. EoE pathophysiology involves esophageal dysmotility and narrowing, which, even without dysphagia, can drive FD. Allergen avoidance diets, especially multi‐food restrictions,^17^, ^18^ limit exposure to diverse flavors and textures,^19^ impeding the development of oral‐motor skills in young children and sensory acceptance of different flavors/smells.^11^, ^20^, ^21^ These restrictions and disrupted mealtime interactions also affect social development and contribute to decreased health‐related QoL, stress, anxiety, and depression in caregivers.^11^, ^14^, ^18^, ^21^, ^22^

While FD are commonly reported in EoE, their prevalence, comorbidities, and impact are not well established.^18^, ^20^, ^21^ FD can be classified into nutritional, medical, feeding skill, and psychological dysfunctions,^6^, ^7^ and while guidelines exist for the general pediatric populations with the published consensus on pediatric feeding disorders,^7^, ^8^, ^9^, ^10^, ^11^ there is no agreement on EoE‐specific definitions, and diagnostic criteria are lacking, risking misdiagnosis and mismanagement.

This publication sets out to perform a systematic review of all the available literature on the reported prevalence of FD in children and adolescents (≤18 years) with EoE. The associated comorbidities and impact on the lives and well‐being of patients and their caregivers were also reviewed.

## 2 METHODS

### 2.1 Search strategy (Figure S1)

**FIGURE 1:**
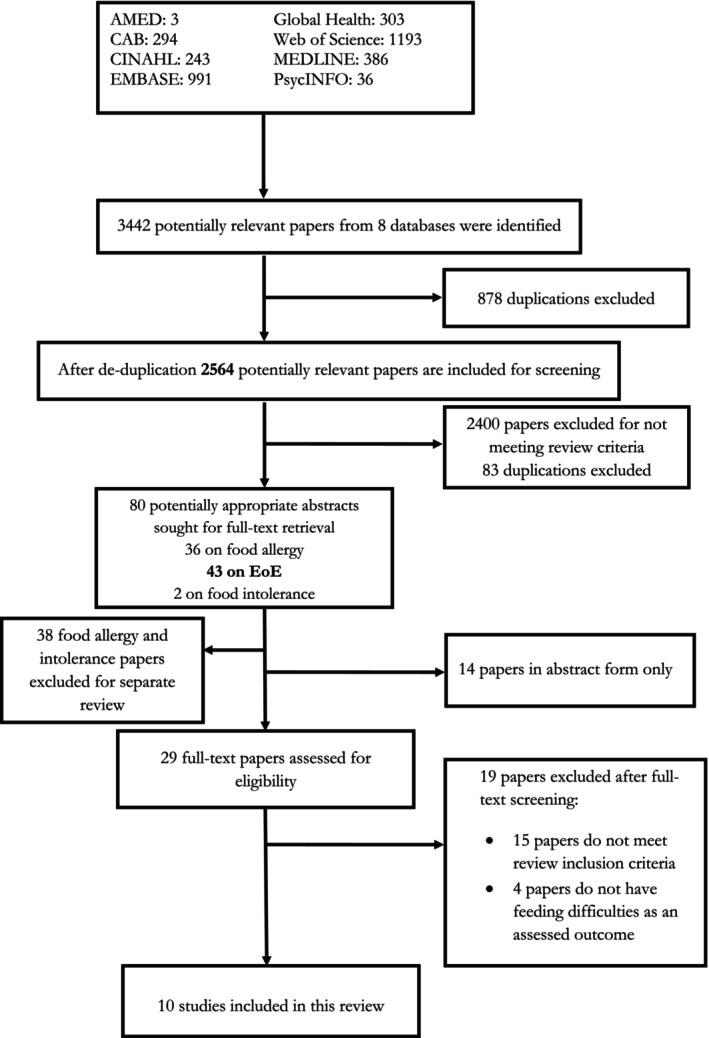
PRISMA flow diagram of screening and selection of studies for qualitative analysis.^23^ PRISMA, Preferred Reporting Items for Systematic Reviews and Meta‐analyses. PRISMA methodology was used to guide the reporting of this systematic review.

This systematic review followed the updated PRISMA guideline^23^ (Figure 1) and was registered with PROSPERO: http://www.crd.york.ac.uk/prospero/ (CRD42022338649).

Relevant articles were identified by searching electronic databases from inception until March 2024: AMED, CAB International, CINAHL, EMBASE, Global Health, ISI Web of Science, MEDLINE, Psych INFO, and international conference proceedings (ISI Conference Proceedings Citation Index, ZETOC‐British Library). Table S4 details the MEDLINE and EMBASE search strategies, adapted for other databases. Additional references were identified via snowballing and by consulting international experts for unpublished or ongoing studies. No language or publication year restrictions applied.

### 2.2 Study eligibility

The study eligibility criteria were designed using the PICOS^24^ framework.

### 2.3 Population

Studies included children and adolescents (≤18 years) with EoE diagnosed by healthcare professionals with histological confirmation. Publications focusing exclusively on eating disorders (e.g., anorexia nervosa) or organic disorders linked to high FD rates, such as autism spectrum disorder, were excluded.

### 2.4 Interventions/conditions

Publications reporting FD prevalence were included. A customized data extraction sheet (Appendix S1) ensured separate reporting of EoE symptoms, like dysphagia, from FD.

### 2.5 Outcome

The primary outcome was FD prevalence in children with EoE (Table 1). Studies without quantitative prevalence data were excluded. Definitions, diagnostic criteria, and FD impacts on growth, HRQL, mental health, and absenteeism (child/parent) were also reported when available.

### 2.6 Study design

Included study types: randomized‐controlled, non‐randomized, cross‐sectional, case–controlled, cohort, and case series (≥5 cases). Excluded: animal studies, reviews, case reports, abstract‐only studies, and qualitative papers.

### 2.7 Screening of studies

Three reviewers (MR, SH, UN) independently screened abstracts, followed by full texts of potentially relevant articles. Discrepancies were resolved by consensus, with a fourth reviewer (RM) arbitrating disagreements.

### 2.8 Data extraction and reporting

Data were extracted using a customized sheet and independently verified by a second reviewer (MR, SH). Descriptive tables summarized study characteristics.

### 2.9 Quality assessment and risk of bias

Two reviewers (MR, SH) independently assessed methodological quality and risk of bias using the EPHPP tool.^25^ A third reviewer (RM) resolved discrepancies. Studies were graded overall and by components, including study design, selection bias, and outcome assessment.

### 2.10 Data syntheses

Due to data heterogeneity, all analyses were qualitative.

## 3 RESULTS

### 3.1 Search results

A search across eight databases identified 3442 potential papers. After removing duplicates and screening abstracts, 29 papers underwent full‐text screening (Figure 1). Of these, 19 were excluded (Table S1), leaving 10 papers with quantitative data on FD prevalence in children with EoE for analysis (Figure 1).^3^, ^26^, ^27^, ^28^, ^29^, ^30^, ^31^, ^32^, ^33^, ^34^ No interventional studies were found. The 10 observational studies comprised of six retrospective chart reviews,^26^, ^27^, ^29^, ^31^, ^32^, ^34^ two qualitative studies (questionnaires),^28^, ^33^ one cohort,^30^ and one cross‐sectional case–control study.^3^

### 3.2 Quality appraisal of included studies

Critical appraisal using the Effective Public Health Practice Project (EPHPP), rating studies as weak, moderate, and strong, rated five studies as moderate and five as weak^26^, ^27^, ^29^, ^31^, ^32^ (Table S3). Weak studies mainly included issues with study design and data collection methods. Most used a retrospective design,^26^, ^27^, ^29^, ^32^ which risks bias and limits causal inference. Four weak‐rated studies also lacked validation of data collection methods,^27^, ^29^, ^31^, ^32^ reducing the reliability of their findings.

### 3.3 Characteristics of included papers

Table 2 shows the characteristics of the included studies. Six studies^3^, ^26^, ^27^, ^28^, ^29^, ^31^ included pediatric patients from the first months of life to 18 years, two studies^33^, ^34^ included patients up to 12 years of age, and one study^32^ included only infants from 5 months to 2 years of age. Across the 10 studies, the mean age of participants ranged from 1.3^32^ to 11^28^ years of age and the year of publication from 2008^27^ to 2022.^33^

**TABLE 2:** Characteristics of the 10 studies that contain prevalence data on feeding difficulties within EoE children.^3^, ^26^, ^27^, ^28^, ^29^, ^30^, ^31^, ^32^, ^33^, ^34^

First author and year of publication	Study design	Location	Size of study	Median age at time of study (years)	EoE and IgE food allergy (%)	Histological findings at diagnosis (Eo/HPF)	Feeding difficulty terminology	Means of recruitment of patients into the study
Azzano (2019)	Retrospective chart review	France (Lyon)	108	9.5	54	15 to >100	Feeding difficulties	Direct tertiary clinic referral
Ferreira (2008)	Retrospective chart review	Brasil (Porto Alegre—Curitiba)	29	7	NS	20 to >60	Refusal to eat	Direct tertiary clinic referral
Hiremath (2019)	Prospective questionnaire	US (Vanderbilt, Tennessee)	80	11	86	>15	Food responsiveness Desire to drink Emotional over‐eating Enjoyment of food Satiety responsiveness Food fussiness Slowness in eating Emotional under‐eating	Direct tertiary clinic referral
Hirsch (2023)	Retrospective chart review	US (Boston)	42	1.3	38	30–100	Gagging or coughing with feeding Difficulty with progression to pureed or solid foods	Direct tertiary clinic referral
Iwanczak (2011)	Retrospective chart review	Poland	84	NS	26.2	>15	Feeding aversion	10 pediatric GI centers, database search of endoscopies
Kamat (2022)	Qualitatitve study. Literature review followed by patient and caregiver interview	US (Atlanta and NY)	24	6.6	58.3	NS	Avoid specific foods Food refusal Poor eating/not eating enough Difficulty eating Feeding aversion or intolerance	Direct tertiary clinic referral
Mehta (2018)	Prospective cohort study	US (Colorado)	91	4.8	59	≥15	Not enjoying eating Taking >20 min to eat Having poor appetite Preferring to drink rather than eat Trying to negotiate what will be eaten	Direct tertiary clinic referral/advertisements and patients seeking routine dental care
Mukkada (2010)	Retrospective chart review	US (Colorado)	200	34 months	88	>15	Learned maladaptive behaviors Food refusal Low volume/variety of intake Poor acceptance of new foods Spitting food out, Grazing Lack of mealtime structure Requiring prompting to eat Inconsistent patterns of eating	Direct tertiary clinic referral
Spergel (2009)	Prospective and restrospective chart review	US (Philadelphia)	562	6.2	NS	>20	Failure to thrive Feeding difficulties	Direct tertiary clinic referral
Wu (2012)	Case–control study	US (Cincinnati, Ohio)	78	7.6	NS	≥15	BPFAS Scores	Direct tertiary clinic referral/advertisements and patients seeking routine dental care

Abbreviations: BPFAS, Behavioral Pediatric Feeding Assessment Scale; NS, not stated.

Across the 10 publications, 6 different diagnostic tools and 18 different terminologies of FD were used (Table 2). Some of the included studies assessed multiple FD phenotypes and therefore used different terminologies. Four of the included publications used validated FD diagnostic tools,^3^, ^28^, ^30^, ^33^ these being the: Behavioural Paediatric Feeding Assessment Scale (BPFAS)^3^, ^30^ and the Child Eating Behaviour Questionnaire (CEBQ),^28^ and the Pediatric EoE Sign/Symptom Questionnaire (PESQ‐P and PESQ‐C).^33^ Despite both using the BPFAS^3^, ^30^ to diagnose FD, Mehta et al.^30^ used five different terminologies, while Wu et al.^3^ used only one. Hiremath et al.^28^ used eight different outcomes from the CEBQ, and Kamat et al.^33^ used six different outcomes from the PESQ‐P and PESQ‐C to define different FD. Out of the remaining studies included in this review, two used their own criteria^31^, ^34^ and four of the included publications did not state which diagnostic tool was used to diagnose FD.^26^, ^27^, ^29^, ^32^

The majority of the studies included in this analysis were conducted in the United States (7 out of 10), with only one study each from France, Poland, and Brazil. Of the studies that reported ethnicity data (reported in only four of the included studies^3^, ^28^, ^31^, ^33^), the percentage of non‐white participants ranged from 10%^31^ to 41.7%.^33^ The proportion of female participants varied between 9.1%^34^ and 37.5%^33^ across the included studies.

### 3.4 Terminology describing feeding difficulties

Significant variability in terminology to describe FD was observed across the 10 articles reviewed (Table 3). Five papers included 1 terminology to describe FD,^3^, ^26^, ^27^, ^29^, ^31^ one paper included two terminologies,^32^ two papers included five terminologies,^30^, ^33^ and two papers included eight terminologies^28^, ^34^ to define FD. This variability highlights the complexity of characterizing FD in this population. The terms used to describe FD ranged from “refusal to eat”^27^ or “food refusal”^33^, ^34^ to more detailed descriptors such as “food responsiveness,”^28^ “desire to drink”^28^ or “preferring to drink rather than eat”^30^ and “emotional over‐eating or under‐eating”.^28^ Other terms included “enjoyment of food”^28^ (or lack thereof),^30^ “satiety responsiveness”^28^ and “food fussiness”^28^ often referred to as “feeding aversion or intolerance”.^29^, ^33^ Additionally, names such as “slowness in eating”^28^ (taking more than 20 min to eat),^30^ “gagging or coughing with feeding”^32^ and “difficulty with progression to pureed or solid foods”^32^ were mentioned. Some studies also noted broader behavioral patterns, including “poor eating”^33^ or “having poor appetite”^30^ “trying to negotiate what will be eaten”^30^ and “learned maladaptive behaviours”^34^ such as “lack of mealtime structure”^34^ and “inconsistent patterns of eating”.^34^ These FD often led to significant nutritional concerns, reflected in terms like “failure to thrive”,^31^ “low volume/variety of intake”^34^ and “poor acceptance of new foods” used in the reviewed publications.^34^

**TABLE 3:** Terminologies, diagnostic criteria, and prevalences of each reported feeding difficulty.^3^, ^26^, ^27^, ^28^, ^29^, ^30^, ^31^, ^32^, ^33^, ^34^

First author and year of publication	Size of study	Female (%)	Means of diagnosing feeding difficulty	Feeding difficulty terminology	Prevalence of feeding difficulty (%)
Azzano (2019)	108	20.3	NS	Feeding difficulties	65.3
Ferreira (2008)	29	24	NS	Refusal to eat	13
Hiremath (2019)	80	19	CEBQ (Child Eating Behavior Questionnaire) FS‐IS (Feeding/Swallowing Impact on Children's Caregivers Questionnaire)	Fussy eating	31^a^
Hirsch (2023)	42	14	Note in medical record (gagging or coughing with feeding; difficulty with progression to pureed or solid foods)	Maladaptive feeding	43
				Gagging or coughing with feeding and/or Difficulty with progression to pureed or solid foods	67
Iwanczak (2011)	84	24	Note in medical record (Feeding aversion)	Food aversion	32.1
Kamat (2022)	24	37.5	Interviews using their own semistructured interview guide that included open‐ended and targeted follow‐up questions Pediatric Eosinophilic Esophagitis Sign/Symptom Questionnaire (PESQ‐P and PESQ‐C)	Avoid specific foods/Food refusal	75/33.3
				Eating slowly	50
				Small appetite	16.7
				Not eating	16.7
				Food modification required/Need for special diet	41.7/29.2
				Drinking liquids while eating	37.5
				Difficulty eating	1.25
Mehta (2018)	53	28	Validated survey measuring feeding dysfunction (BPFAS)	Feeding difficulties	37
Mukkada (2010)	200	9.1	Protocol developed by the Children's Hospital Colorado	Feeding difficulty^b^	16.5
				Learned maladaptive feeding behaviors^c^	93.9
				Low variety intake/Requiring prompting to eat/Low volume of intake^c^	90.9/87.9/81.8
				Food refusal^c^	87.9
				Poor acceptance of new foods^c^	84.8
				Lack of mealtime structure/Inconsistent patterns of eating^c^	81.8/78.8
				Easily distracted from eating^c^	60.6
				Prolonged feeding times/Holding food in mouth^c^	57.6/27.3
				Spitting food/Grazing^c^	27.3/78.8
Spergel (2009)	562	25	Feeding and swallowing program	“Failure to thrive or feeding difficulties” together as one category	20.9
Wu (2012)	78	22	Validated survey measuring feeding dysfunction (BPFAS)	Feeding difficulties	75.3^d^

Abbreviations: BPFAS, Behavioral Pediatric Feeding Assessment Scale; NS, not stated.

^a^ Prevalence is non‐significantly higher than the control group (34%).

^b^ They used this term to encompass all feeding difficulties observed in the entire EoE cohort. These are separated out into the other terminologies.

^c^ Percentages refer to how many subjects present each feeding difficulty separately, out of the total number of children with FD.

^d^ Diagnostic scores were significantly higher than in the control group.

### 3.5 Prevalence data

The 10 included studies^3^, ^26^, ^27^, ^28^, ^29^, ^30^, ^31^, ^32^, ^33^, ^34^ reported the prevalence of FD in children with EoE. Out of the included studies using validated diagnostic tools,^3^, ^28^, ^30^, ^33^ the prevalence of FD ranged from 16.7%^33^ to 75.3%.^3^ The highest prevalence of 75.3%,^3^ was recorded using the BPFAS in a retrospective study in the United States, while Mehta et al.^30^ reported 37% prevalence using the same tool in a prospective study in the United States.

Five of the included papers were retrospective chart reviews, with patient records sourced directly from tertiary clinics.^26^, ^27^, ^29^, ^32^, ^34^ Across these, the reported frequency of FD ranged from 13%^27^ to 67%.^32^ Four papers assessed FD only by parental report,^3^, ^28^, ^30^, ^33^ with a prevalence ranging from 37% to 75.3%; and three studies were supported by medical records,^29^, ^32^, ^34^ reporting 16.5%^34^ to 67%^32^ prevalence.

### 3.6 Comparative data

Only two studies included a comparative group. One such study^28^ compared quantitative data regarding specific phenotypes of feeding difficulty, as well as EoE‐specific symptoms, between EoE patients and healthy controls. In this study, the CEBQ tool was used and recorded 31% of EoE patients as fussy eaters (control 34%), and 24% as being slow eaters (control 9%), neither of which reached statistical significance. The second such study, by Wu et al.,^3^ used the BPFAS tool to compare patients with eosinophilic gastrointestinal diseases (EGID)—of which 85% had EoE—with a gender‐ and aged‐matched healthy control group. While no specific comparison was made for only those with EoE, children with EGID exhibited significantly higher scores on the BPFAS compared to healthy controls, which, in turn, was associated with higher parental stress and parental maladjustment of eating dysfunction.

### 3.7 Dietary management

Seven^26^, ^27^, ^28^, ^29^, ^30^, ^31^, ^32^ of the 10 articles included the percentage of patients managed with elimination diets, which ranged from 24% to 84%.^27^, ^29^ Only four^27^, ^30^, ^31^, ^32^ of these studies included information on specific foods that were avoided in their diet because of EoE. Cow's milk and/or red meat was avoided by 24% of the EoE population in the publication by Ferreira et al.,^27^ while Hirsch et al.,^32^ reported 17% of their EoE population followed a strict cow's milk elimination diet, and 9.5% followed an amino acid formula diet with multiple food restrictions. Mehta et al.^30^ reported 38% of their patients with EoE were being treated with food allergen restriction diets at the time of enrolment, with the most commonly avoided foods being egg, peanut, tree nuts, dairy, soy, fish, wheat, and corn. The only included publication^31^ to report the percentages of patients avoiding each food group reported the most frequent food restrictions to be the following: cow's milk (17%), egg (11%), wheat (9.6%), soy (7.8%), corn (7.8%), beef (6.6%), chicken (6.1%), peanut (5.4%), potato (4.8%), and rice (4.1%).

### 3.8 Concomitant IgE‐mediated food allergy/sensitization

Seven^26^, ^28^, ^29^, ^30^, ^32^, ^33^, ^34^ of the 10 included papers reported the prevalence of concomitant IgE‐mediated allergies/sensitization (not challenge proven) in the EoE study population, which ranged between 38%^32^ and 88%^31^ (Table 4). A further publication^29^ recorded that 26.2% of their EoE population also had food allergy/sensitization, but the food allergy/sensitization type was not described. While Mukkada et al.^34^ reported the highest prevalence of concomitant IgE‐mediated food allergy/sensitization, the prevalence of FD was recorded to be 16.5%, one of the lowest in this systematic review. There therefore does not seem to be any analyzable trend in regard to food allergy/sensitization and FD prevalence across these seven publications.

**TABLE 4:** Prevalence of IgE allergy and EoE.^3^, ^26^, ^27^, ^28^, ^29^, ^30^, ^31^, ^32^, ^33^, ^34^

First author and year of publication	Size of study	EoE and IgE food allergy (%)	Confirmed IgE food allergy?	Other food allergy‐related comorbidities in addition to EoE (%)
Azzano (2019)	108	54.0	61 (56.4%) had confirmation by food SPT and 47 (43.5%) by sIgE	AD 39.0 Asthma 61.8 Rhinitis 51.0
Ferreira (2008)	29	NS	NS	NS
Hiremath (2019)	80	86.0	NS	AD 8.0
Hirsch (2023)	42	38.0	NS	AD 69.0
Iwanczak (2011)	84	26.2^a^	NS if food allergy is IgE or non‐IgE	AD 7.1 Asthma 17.8 Rhinitis 9.5
Kamat (2022)	24	58.3	NS	AD 12.5 Asthma 45.8 Rhinitis 16.7
Mehta (2018)	53 (31 with EoE)	59.0^b^	Confirmed by food sIgE on 100%	AD 30.0 Seasonal allergies 26.0
Mukkada (2010)	200	88.0	Confirmed by food SPT and/or sIgE on 100%	In those with a FD: 52.0% had eczema, allergic rhinitis, or asthma
Spergel (2009)	562	NS	NS	AD 12.58 Asthma 37.5 Allergic rhinitis 39.19
Wu (2012)	78	NS	NS	Eosinophilic gastroenteritis 15.0

Abbreviations: AD, atopic dermatitis; FD, feeding difficulties; NS, not stated; SPT, skin prick test.

^a^ Not stated if food allergy was IgE o non‐IgE.

^b^ IgE food allergy in 59% of the 31 included patients with EoE.

In addition, none of the aforementioned seven publications recorded confirmation of IgE‐mediated allergy via oral food challenge, nor comparative data on the prevalence of FD within this subset of patients with concomitant IgE‐mediated food allergy/sensitization and EoE. One of the seven papers^30^ reported concomitant IgE‐mediated food allergy/sensitization based on sIgE levels alone, and a further two publications reported IgE‐mediated food allergy/sensitization based on either the skin prick test or sIgE level to the food allergen; none of which referred to the history of previous reactions. The remaining four papers did not state any information as to how the diagnosis of the reported IgE‐mediated food allergy/sensitization had been established.

### 3.9 Growth and nutritional impact

Five of the studies^26^, ^27^, ^29^, ^32^, ^34^ included data on the impacts of EoE on certain growth parameters or on nutritional status, ranging from 15.7% to 33%, but did not include data on the impact of FD on the aforementioned.

Iwanczak et al.,^29^ observed 22.6% of children having malnutrition (defined as below the third percentile), with the highest percentage among children aged 1–6 years (27.2%), and decreasing with increasing age. Only three studies^30^, ^32^, ^34^ reported information on patients' weight and height parameters. One study reported unaffected growth^30^ using body mass index (BMI) and weight‐for‐height *z*‐score. Another reported the average BMI and height‐for‐age *z*‐score within the EoE study population to be lower than the average of the general population,^34^ while Hirsch et al.^32^ reported a higher than average weight‐for‐age.^32^

Mukkada et al.,^34^ reported 9% of patients presented with mental health issues (depression or anxiety). Parental stress, using an unvalidated questionnaire, was also reportedly higher in those children with EoE affected by FD compared to those withoutFD.^34^ Hiremath et al.^28^ investigated the impact of the FD on the caregivers' health‐related QoL using the FS‐IS questionnaire.^35^ Compared to controls, the EoE group found it challenging to make plans to eat out (*p* < .001), to feed their child due to the time required to prepare food (*p* < .001) and to receive differing opinions from family or professionals (*p* < .001). Additionally, while not statistically significant (*p* = .55), they also reported more difficulties feeding their children due to a lack of information on how to feed them like other children. The EoE group also had greater concern for breathing and choking while feeding (*p* = .02). The odds were 44.16 times higher for males and 49.21 times higher for those with food allergies.

## 4 DISCUSSION

This systematic review, to the best of our knowledge, is the first comprehensive investigation of the prevalence and any potential associations and impact of FD in children with EoE. Reported FD prevalence ranged from 13% to 75.3%, using 18 different terminologies and six diagnostic tools across 10 studies.

The existing literature predominantly comprised of retrospective and cross‐sectional studies, with there being only two prospective studies meeting the inclusion criteria for this systematic review. Retrospective study designs are inherently limited in their ability to establish a clear chronological relationship between the onset of EoE and feeding difficulty. Additionally, they do not provide insight into the potential transient nature of the FD symptoms, as it is often reported in healthy children, leaving a gap in understanding whether the reported FD persists over time. Regarding the quality assessment of the included studies, the methodological weaknesses underline the need for cautious interpretation of the results and highlight areas for improvement in future research. The lack of prospective studies and the high variability in diagnostic terms, alongside the absence of standardized definitions across studies, impairs the ability to compare data from different studies. This wide range of terms underscores the need for standardized language to effectively identify and manage FD in children with EoE. Recent publications on pediatric FD have proposed using the criteria for pediatric feeding disorder^11^ (present in ICD 10^36^) as universal criteria for children, but there has been no study to date that has used these criteria and validated its use in EoE or any other allergic disorders. Furthermore, there are symptoms that are unique to EoE, which may not be captured by such a generic criterion.

Despite the known importance of dietary management in EoE, only four studies included information on whether elimination diets were being followed by participants, with only one^32^ including detailed information on the specific foods avoided or type of diet followed by participants. None of the aforementioned provided information on whether the patients with food restrictions also had FD. Iwanczak et al.,^29^ reported a predominance in FD and malnutrition in children younger than 6 years old.

This lack of data presents a significant gap in our understanding of how early dietary interventions, including the number and types of foods avoided, might influence the course of EoE and the development of FD, for instance, through limited exposure to different textures and tastes. Understanding such associations could be pivotal in identifying dietary factors associated with FD and optimizing dietary management strategies for patients with concurrent EoE and FD.

Given the data available, we could not establish if having IgE‐mediated food allergy in the context of EoE influences the rate of FD. To address these methodological limitations, future studies should analyze how different forms of confirmed food allergy as well as EoE can influence the development of FD. These studies should include standardized diagnostic methods, including food challenges, for these conditions, and harmonized assessment tools for FD should be used.

The demographic analysis of the included studies reveals a predominance of Caucasian males with EoE. Despite this being in keeping with the findings of a recent systematic review of the literature on the demographics of EoE diagnoses,^37^ this demographic bias, however, raises concerns about the generalizability of the findings, as the disease presentation and response to treatment may differ across different racial and gender groups.^38^ In addition, seven out of the 10 included publications in this systematic review were conducted in the United States. This geographic concentration limits the applicability of the findings to other regions, where dietary habits, healthcare systems, and genetic backgrounds may differ significantly. The lack of region‐specific data poses challenges in translating these findings into clinical practice outside of the United States.

Information on on‐going supportive interventions such as dietitian access was also not included in any of the publications. Therefore, the potential effects such support may have had on the development and/or persistence of FD also cannot be assessed. The amplified state of vigilance by carers to ensure the avoidance of certain foods may influence the reporting and subsequent diagnosis of FD as what may be regarded as dysfunctional feeding may actually be a necessary adaptation to living with EoE.

Moreover, information on the severity of EoE or the specific FD symptoms, which is a crucial factor in understanding the clinical impact of these conditions, is lacking in the included publications. A recent study by Chebar‐Lozinsky et al.^39^ in children with non‐IgE‐mediated food allergy reported that the number of foods eliminated was not associated with FD, but the age and the severity of symptoms were. The omission of a marker of severity may therefore limit the ability to evaluate the full spectrum of disease burden and the effectiveness of various treatment approaches. Nevertheless, the data generated from this systematic review does imply that FD is commonly reported in children with EoE. The range reported in this systematic review overlaps somewhat with that of the recent systematic review of FD in children with IgE‐mediated food allergy, of 13.6%–40%.^2^

### 4.1 Limitations

This systematic review has several limitations. Most notably, the conclusions are limited by methodological heterogeneity and the limited number of eligible studies. Therefore, we propose a series of practical recommendations for conducting high‐quality studies on EoE and FD (Table S2).

Comparing data across countries is difficult due to geographic variations in EoE prevalence,^40^ differences in eating habits, parenting styles, and healthcare systems. Most studies included predominantly Caucasian, male samples and were conducted in the United States.

Key factors potentially influencing FD, such as diagnostic delays,^41^ symptom severity,^39^ number of eliminated foods,^42^, ^43^ and treatment type, were not reported, adding to the limitations and heterogeneity of findings. Additionally, most studies were retrospective or cross‐sectional, lacking clarity on the chronology of EoE onset and FD or whether these difficulties were transient or persistent.

### 4.2 Strengths of the study

The comprehensive review of eight international electronic databases with high methodological rigor increases the strength of the conclusions of this systematic review.

## 5 CONCLUSION

This systematic review supports the clinical observation that FD are commonly reported in children with EoE, with prevalences ranging from 13%^27^ to 75.3%.^3^ Great heterogeneity in definitions and diagnostic criteria was identified. A total of 18 different terms to define FD and 6 diagnostic tools were utilized in the 10 included studies. Four of the publications did not specify which diagnostic tool was used to diagnose FD,^26^, ^27^, ^29^, ^32^ and two of them used their own protocols. A high prevalence of concomitant EoE and IgE‐mediated food allergy/sensitization in the study populations was observed across studies, ranging from 26.2%^29^ to 88%^34^ and only one paper reported the number of foods being avoided by participants. In addition, the lack of prospective studies hinders assessing whether FD might be transient or pre‐existing to the EoE diagnosis.

Therefore, while significant strides have been made in understanding EoE and FD, there remain gaps in our knowledge. Given the increasing prevalence of EoE,^37^ this highlights the need for consensus‐based definitions and diagnostic tools for FD in EoE to ensure early recognition and optimal management by multidisciplinary teams.

This EAACI Task Force aims to conduct a Delphi Consensus exercise to reach agreement on which tools and terminology should be used to assess FD in children with EoE. Future research should aim to address these gaps, with a focus on prospective long‐term studies, standardized terminology, and a more comprehensive understanding of dietary management in these patients. This will allow for a better understanding of the potential underlying pathologic mechanisms and risk factors linking EoE to the development of FD.

## AUTHOR CONTRIBUTIONS

**Maria Ruano‐Zaragoza:** Conceptualization; investigation; funding acquisition; writing – original draft; writing – review and editing; visualization; validation; methodology; software; formal analysis; project administration; resources; supervision; data curation. **Sarah‐Anne Hill:** Conceptualization; investigation; funding acquisition; writing – original draft; writing – review and editing; visualization; validation; methodology; software; formal analysis; project administration; data curation; supervision; resources. **Ulugbek Nurmatov:** Supervision; conceptualization; investigation; methodology; validation; data curation; formal analysis; software. **Imke Reese:** Supervision; validation; investigation. **Mario C. Vieira:** Supervision; investigation; validation. **Christophe Dupont:** Supervision; validation; investigation. **Carina Venter:** Supervision; validation; investigation. **Joanne Walsh:** Supervision; investigation; validation. **Glauce Yonamine:** Supervision; validation; investigation. **Alexia Beauregard:** Supervision; investigation; validation. **Fernanda González‐Matamala:** Supervision; validation; investigation. **Antonella Cianferoni:** Supervision; validation; investigation. **Audrey DunnGalvin:** Supervision; validation; investigation. **Marta Vazquez‐Ortiz:** Supervision; resources; data curation; methodology; validation; visualization; writing – review and editing; funding acquisition; investigation; conceptualization; writing – original draft; project administration. **Rosan Meyer:** Supervision; conceptualization; investigation; funding acquisition; methodology; validation; visualization; writing – review and editing; resources; data curation; writing – original draft; project administration.

## FUNDING INFORMATION

This Systematic review was supported by the European Academy of Allergy and Clinical Immunology (EAACI) under the EAACI taskforce Feeding difficulties in children with food allergies/Pediatrics/2022.

## CONFLICT OF INTEREST STATEMENT

Mario C. Vieira: personal fees as a consultant and/or speaker from Danone Nutricia, Nestlé Nutrition Institute, and Aché Laboratories, outside the submitted work. Alexia Beauregard: personal fees as a speaker for Abbott. Imke Reese: personal fees as a speaker from Danone Nutricia and Nestlé Nutrition Institute, outside the submitted work. Dr. Carina Venter: reports grants from Reckitt Benckiser, grants from Food Allergy Research and Education, and grants from National Peanut Board during the conduct of the study; and personal fees from Reckitt Benckiser, Nestlé Nutrition Institute, Danone, Abbott Nutrition, Else Nutrition, and Before Brands, outside the submitted work. Dr. Rosan Meyer: personal fees as a consultant and/or speaker from Danone Nutricia, Nestlé Nutrition Institute, Abbott Nutrition, and Reckitt Benckiser; and grants from Danone/Nutricia, and consultancy from Else Nutrition. Christophe Dupont: personal fees as a consultant and/or speaker from Danone, Nestlé, Abbott, Biostime, and DBV Technologies, outside the submitted work.

### PEER REVIEW

The peer review history for this article is available at https://www.webofscience.com/api/gateway/wos/peer‐review/10.1111/pai.70087.

## Supporting information


Data S1
