# Supplementary material for: Systematic review of feeding difficulties in children with eosinophilic esophagitis: An EAACI Task Force report
Source: Pediatr Allergy Immunol. 2025 Apr 17;36(4):e70087. doi: 10.1111/pai.70087 (PMC12004433; doi:10.1111/pai.70087)
Supplement: Supplementary file 1 — Data S1 [file PAI-36-e70087-s001.docx]

**SUPPLEMENTARY MATERIAL**

**Figure S1.** Flow diagram outlining the methodology of this systematic review.


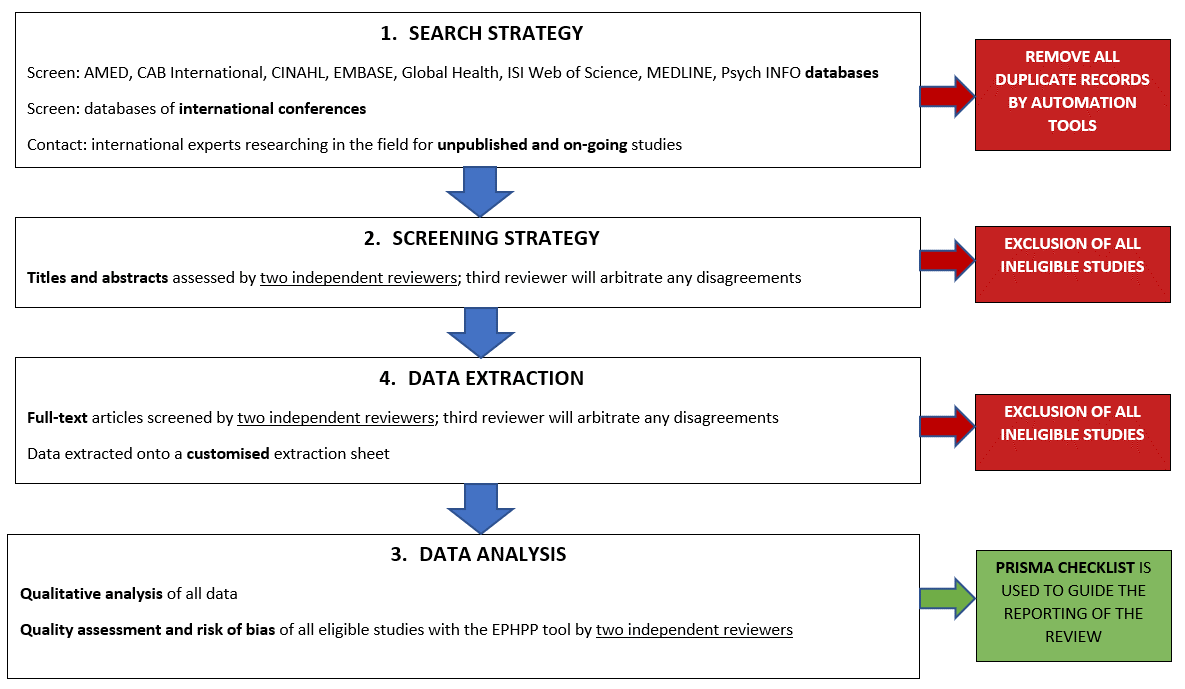


**Table S1**. Table showing the twenty-nine excluded papers, in Vancouver referencing format, after full-text screening and the reasons why^1-29^

| **PAPER** | **REASON FOR EXCLUSION** |
| --- | --- |
| Barfjani, S. H., et al. (2016). "Esophagitis in infants and toddlers with feeding difficulties: Prevalence and associated clinical characteristics." Journal of Pediatric Gastroenterology and Nutrition **63**(Supplement 2): S18-S19. | Unobtainable |
| Beser, O. F., et al. (2011). "Evaluation of 20 cases who have eosinophilic esophagitis." Acta Paediatrica, International Journal of Paediatrics **46**(3): 42. | Does not meet inclusion criteria (abstract only) |
| Guarnere, S., et al. (2020). "Speech-language pathologists promote management of children with eosinophilic esophagitis." American Journal of Gastroenterology **115**(SUPPL): S609 | Does not meet inclusion criteria (poster) |
| Hilow, E., et al. (2019). "Natural history of eosinophilic esophagitis in children: A retrospective chart review from a tertiary care center." Journal of Pediatric Gastroenterology and Nutrition. Conference: North American Society for Pediatric Gastroenterology, Hepatology and Nutrition Annual Meeting, NASPGHAN **69**(Supplement 2). | Does not meet inclusion criteria (abstract only) |
| Hoofien, A., et al. (2019). "Pediatric Eosinophilic Esophagitis: Results of the European Retrospective Pediatric Eosinophilic Esophagitis Registry (RetroPEER)." Journal of Pediatric Gastroenterology and Nutrition **68**(4): 552-558. | Feeding difficulties not an assessed outcome |
| Levin, M. and C. Motala (2011). "Eosinophilic esophagitis in Cape Town, South Africa." Clinical and Translational Allergy. Conference: Food Allergy and Anaphylaxis Meeting **1**(SUPPL. 1). | Does not meet inclusion criteria (abstract only) |
| Menzies, J., et al. (2017). "Prevalence of malnutrition and feeding difficulties in children with esophageal atresia." Journal of Pediatric Gastroenterology and Nutrition **64**(4): e100-e105. | Does not meet inclusion criteria (focused on feeding difficulties in children with esophageal atresia, not EoE) |
| Pham, A., et al. (2019). "Feed-easy: Feeding disorders in children with esophageal atresia study." Diseases of the Esophagus **32**(Supplement 1): 8-9. | Does not meet inclusion criteria (abstract only) |
| Taylor, J. and E. Volonaki (2019). "Incidence of eosinophilic oesophagitis in a UK paediatric population over a 10-year period." Journal of Pediatric Gastroenterology and Nutrition **68**(Supplement 1): 313. | Unobtainable |
| Brytek-Matera, A., et al. (2022). "Symptoms of Avoidant/Restrictive Food Intake Disorder among 2-10-Year-Old Children: The Significance of Maternal Feeding Style and Maternal Eating Disorders." Nutrients **14**(21): 13. | Feeding difficulties in EoE patients not an assessed outcome |
| Chudoba, A. (2022). "Eosinophilic gastrointestinal diseases in childhood." Pediatria I Medycyna Rodzinna-Paediatrics and Family Medicine **18**(2): 119-124. | Unobtainable |
| Ciciulla, D., et al. (2023). "Systematic Review of the Incidence and/or Prevalence of Eating Disorders in Individuals With Food Allergies." The Journal of Allergy & Clinical Immunology in Practice **11**(7): 2196-2207.e2113. | Does not meet inclusion criteria (not a primary research) |
| Cifra, N. and J. Lomas (2022). "158. Case Series: Eosinophilic Esophagitis Presenting as an Eating or Feeding Disorder." Journal of Adolescent Health **70**(4 Supplement): S83. | Does not meet inclusion criteria (poster) |
| Cifra, N. and J. M. Lomas (2022). "Differentiating Eosinophilic Esophagitis and Eating/Feeding Disorders." Pediatrics **149**(4): 01. | Does not meet inclusion criteria (only 4 cases, does not qualify for case series) |
| Ciurez, B. T., et al. (2023). "Risk Factors Related to Eating Disorders in a Romanian Children Population." Nutrients **15**(13): 10. | Feeding difficulties in EoE patients not an assessed outcome |
| Dumont, E., et al. (2022). "Feeding/Eating Problems in Children Who Refrained From Treatment in the Past: Who Did (Not) Recover?" Frontiers in Pediatrics **10** (no pagination). | Feeding difficulties in EoE patients not an assessed outcome |
| Hollaender, M., et al. (2022). "The incidence of eosinophilic oesophagitis in 2007-2017 among children in North Denmark Region is lower than expected." BMC Pediatrics **22**(1) (no pagination). | Does not meet inclusion criteria (focused on incidence of EoE, not feeding difficulties) |
| Keles, M. N., et al. (2023). "Oropharyngeal Dysphagia in Children with Eosinophilic Esophagitis." Dysphagia **38**(1): 474-482. | Does not meet inclusion criteria (its focus is on the swallow and risk assessment) |
| Koenigsberg, R., et al. (2022). "Body mass index in relation to presenting symptoms and age upon diagnosis of Eosinophilic Esophagitis." Journal of Allergy and Clinical Immunology **149**(2 Supplement): AB205. | Does not meet inclusion criteria (conference abstract) |
| Lucendo, A. J., et al. (2022). "EoE CONNECT, the European Registry of Clinical, Environmental, and Genetic Determinants in Eosinophilic Esophagitis: rationale, design, and study protocol of a large-scale epidemiological study in Europe." Therapeutic Advances in Gastroenterology **15**(no pagination). | Does not meet inclusion criteria (feeding difficulties not mentioned) |
| Monteiro, A., et al. (2023). "How maternal personality traits can affect children's neophobia to milk and egg food allergies?" Allergy: European Journal of Allergy and Clinical Immunology **78**(Supplement 111): 484-485. | Does not meet inclusion criteria (conference abstract) |
| Moroco, A. E. and N. L. Aaronson (2022). "Pediatric Dysphagia." Pediatric Clinics of North America **69**(2): 349-361. | Does not meet inclusion criteria (review) |
| Muir, A. B., et al. (2023). "It takes a village to manage eosinophilic esophagitis." Annals of Allergy, Asthma and Immunology **130**(1): 13-14. | Does not meet inclusion criteria (review) |
| Patrawala, M. M., et al. (2022). "Avoidant-restrictive food intake disorder (ARFID): A treatable complication of food allergy." Journal of Allergy and Clinical Immunology: In Practice **10**(1): 326-328.e322. | Does not meet inclusion criteria (not EoE, focus is on other disorders) |
| Philpott, H., et al. (2022). "Patient demographics and management of eosinophilic esophagitis in Australasian children: Racial differences and regional patterns of care vary across all capital cities in a decade of data." Journal of Gastroenterology and Hepatology **37**(Supplement 1): 211. | Unobtainable |
| Rodrigues, V. C. C., et al. (2022). "Feeding difficulties in children fed a cows' milk elimination diet." British Journal of Nutrition **128**(6): 1190-1199. | Does not meet inclusion criteria (not focused on EoE) |
| Sitarik, A. R., et al. (2021). "Infant Feeding Practices and Subsequent Dietary Patterns of School-Aged Children in a US Birth Cohort." Journal of the Academy of Nutrition and Dietetics **121**(6): 1064-1079. | Does not meet inclusion criteria (not focused on EoE) |
| Votto, M., et al. (2022). "Eosinophilic esophagitis an update in children." Acta Bio-Medica de l Ateneo Parmense **93**(S3): e2022034. | Does not meet inclusion criteria (review) |
| Yong, C., et al. (2023). "Parental food neophobia, feeding practices, and preschooler's food neophobia: A cross-sectional study in China." Appetite **185** (no pagination). | Does not meet inclusion criteria (not focused on EoE) |

**Table S2.** Minimal Requirements for Conducting High-Quality Studies on EoE and Feeding Difficulties

| **Aspect** | \| **Recommendation** \| \| --- \| |
| --- | --- | --- |
| \| Terminology \| \| --- \| | \| Use consistent and standardized definitions of feeding difficulties. \| \| --- \| |
| \| Study design \| \| --- \| | \| Prospective designs to reduce bias and improve data quality. \| \| --- \| |
| \| Diagnostic tools \| \| --- \| | \| Employ validated tools for assessing feeding difficulties, and for diagnosing underlying conditions (EoE, food allergy) \| \| --- \| |
| \| Population \| \| --- \| | \| Clearly define inclusion/exclusion criteria, including EoE severity. \| \| --- \| |
| \| Outcomes \| \| --- \| | \| Report on nutritional, psychological, and social impacts systematically. \| \| --- \| |

**Table S3.** Critical appraisal of included studies assessed by the Effective Public Health Practice Project (EPHPP).**^30^**

*Abbreviations: EPHPP - the Effective Public Health Practice Project. S – Strong. M – Moderate. W – Weak.*

| **Study** | **Design** | **Selection Bias** | | | **Study Design** | | | **Confounders** | | | **Blinding** | | | **Data Collection Method** | | | **Withdrawals and Dropouts** | | | **Global Rating** | | |
| --- | --- | --- | --- | --- | --- | --- | --- | --- | --- | --- | --- | --- | --- | --- | --- | --- | --- | --- | --- | --- | --- | --- |
|  |  | **S** | **M** | **W** | **S** | **M** | **W** | **S** | **M** | **W** | **S** | **M** | **W** | **S** | **M** | **W** | **S** | **M** | **W** | **S** | **M** | **W** |
| **Azzano 2019** | **Retrospective chart review** |  | X |  |  |  | X |  |  | X |  | X |  |  | X |  |  | X |  |  |  | X |
| **Ferreira 2008** | **Retrospective chart review** |  | X |  |  |  | X |  |  | X |  | X |  |  |  | X |  | X |  |  |  | X |
| **Hiremath 2019** | **Cohort analytic** |  | X |  |  | X |  |  | X |  |  | X |  | X |  |  |  | X |  |  | X |  |
| **Hirsch 2023** | **Retrospective chart review** |  | X |  |  |  | X |  | X |  |  | X |  |  |  | X |  | X |  |  |  | X |
| **Iwanczak 2011** | **Retrospective chart review** |  | X |  |  |  | X |  | X |  |  | X |  |  |  | X |  | X |  |  |  | X |
| **Kamat 2022** | **Questionnaire** |  | X |  |  | X |  |  | X |  |  | X |  | X |  |  |  |  | X |  | X |  |
| **Mehta 2018** | **Cohort** |  | X |  |  | X |  |  | X |  |  | X |  | X |  |  |  |  | X |  | X |  |
| **Mukkada 2010** | **Retrospective chart review** |  | X |  |  |  | X |  | X |  |  | X |  |  | X |  |  | X |  |  | X |  |
| **Spergel 2009** | **Chart review** |  | X |  |  |  | X |  | X |  |  | X |  |  |  | X |  | X |  |  |  | X |
| **Wu 2010** | **Cross-sectional case-control** |  | X |  |  | X |  |  | X |  |  | X |  | X |  |  | X |  |  |  | X |  |

**Table S4.** Search strategy, MEDLINE format:

1. exp Food Hypersensitivity /
2. exp Milk Hypersensitivity/
3. exp Egg Hypersensitivity/
4. exp Peanut Hypersensitivity/
5. exp Tree nut Hypersensitivity/
6. exp Nut Hypersensitivity/
7. exp Wheat Hypersensitivity/
8. ((food or milk or egg or peanut or arachis hypogaea or tree nut or hazelnut or brazil nut or walnut or chestnut or pistachio or almond or legumes or wheat or rice or soy or fish or seafood or shellfish or shrimp or lobster or crab or crawfish or kiwi or apple or peach or apricot or cherry or pear or plum or tomato or green pea or potato or carrot or parsley or celery or additives) adj3 (allerg* or hypersensitivit*)).mp.
9. exp Food allergy/
10. allergic reaction to food.mp.
11. IgE-mediated food allergy.mp
12. Non-IgE-mediated food allergy.mp.
13. Eosinophilic esophagitis.mp.
14. or/1-13
15. "Feeding and Eating Disorders"/ or behavioural feeding difficulties.mp. or Feeding Behavior/
16. "Feeding and Eating Disorders of Childhood"/ or avoidant restrictive food intake disorder.mp. or Avoidant Restrictive Food Intake Disorder/
17. ARFID.mp.
18. eating disorder.mp.
19. **food neophobia.mp**.
20. food refusal.mp.
21. (**food intolerance** or aversive eating or aversive feeding or food avoidance or **picky eating** or **picky fussy eating** or **fussy eating**).mp.
22. or/15-21
23. exp Child/
24. Children.mp.
25. (pediatri* or paediatri*).mp.
26. (boy* or girl* or new born or new-born or infant* or baby or toddler or child or kid*).mp.
27. or/23-26
28. 14 and 22 and 27
29. (advertisements or animation or architectural drawings or bibliography or biography or book illustrations or bookplates or charts or comment or letter or editorial or news or patient education handout or published erratum or retraction of publication).mp.
30. 28 not 29

**Search strategy and results, MEDLINE format:**

| Ovid MEDLINE(R) <1946 to April Week 3 2022> | |  |
| --- | --- | --- |
| **#** | **Searches** | **Results** |
| 1 | exp Food Hypersensitivity/ | 22716 |
| 2 | exp Milk Hypersensitivity/ | 2787 |
| 3 | exp Egg Hypersensitivity/ | 870 |
| 4 | exp Peanut Hypersensitivity/ | 1597 |
| 5 | exp Tree nut Hypersensitivity/ | 436 |
| 6 | exp Nut Hypersensitivity/ | 436 |
| 7 | **exp Wheat Hypersensitivity/** | 573 |
| 8 | ((food or milk or egg or peanut or arachis hypogaea or tree nut or hazelnut or brazil nut or walnut or chestnut or pistachio or almond or legumes or **wheat** or rice or soy or fish or seafood or shellfish or shrimp or lobster or crab or crawfish or kiwi or apple or peach or apricot or cherry or pear or plum or tomato or green pea or potato or carrot or parsley or celery or additives) adj3 (allerg* or hypersensitivit*)).mp. | 27872 |
| 9 | exp Food allergy/ | 22716 |
| 10 | allergic reaction to food.mp. | 31 |
| 11 | IgE-mediated food allergy.mp. | 515 |
| 12 | Non-IgE-mediated food allergy.mp. | 108 |
| 13 | Eosinophilic esophagitis.mp. | 2910 |
| 14 | or/1-13 | 30278 |
| 15 | "Feeding and Eating Disorders"/ or **behavioural feeding difficulties**.mp. or Feeding Behaviour/ | 17425 |
| 16 | "Feeding and Eating Disorders of Childhood"/ or avoidant restrictive food intake disorder.mp. or Avoidant Restrictive Food Intake Disorder/ | 818 |
| 17 | ARFID.mp. | 157 |
| 18 | eating disorder.mp. | 11490 |
| 19 | **food neophobia.mp.** | 312 |
| 20 | food refusal.mp. | 363 |
| 21 | (**food intolerance** or aversive eating or aversive feeding or food avoidance or **picky eating or picky fussy eating or fussy eating**).mp. | 1655 |
| 22 | or/15-21 | 25549 |
| 23 | exp Child/ | 2066792 |
| 24 | Children.mp. | 1031619 |
| 25 | (pediatri* or paediatri*).mp. | 413285 |
| 26 | (boy* or girl* or new born or new-born or infant* or baby or toddler or child or kid*).mp. | 3709820 |
| 27 | or/23-26 | 3869110 |
| 28 | 14 and 22 and 27 | 352 |
| 29 | (advertisements or animation or architectural drawings or bibliography or biography or book illustrations or bookplates or charts or comment or letter or editorial or news or patient education handout or published erratum or retraction of publication).mp. | 2400739 |
| **30** | **28 not 29** | **338** |

**Appendix S1.** Data Extraction headings, copied from the customised data extraction sheet (Excel):

Study information

1st author

year

study design

study duration

country

Participant information

total no. whole study (intervention group)

no. females

% of females

% white ethnicity

means of recruitment into study: primary centre referral, tertiary centre referral, direct tertiary clinic recruitment, advertisement/outreach (1-4)

EoE only/ EoE and IgE allergy (%)

Food allergy-related comorbidities in addition to EoE (%, 1.IgE mediated food allergy, 2. FPIES, 3. protein-induced enteropathy, 4. Proctocolitis, 5. Other non igE mediated food allergy, 6. Atopic dermatitis, 7. Other (%)

Endoscopic findings at diagnosis

endoscopic reference score (yes -indicate which one, no, not mentioned)

Histological confirmation of EoE diagnosis (yes / no)

Histological findings at diagnosis (number of eos/HPF and other features)

Medications used for EoE (1 - PPIs, 2- steroids, 3- dietary intervention, 4 - other (% of participants on each)

number/% had SPT/sIgE tests

Foods avoided due to EoE: egg, milk, wheat, soya, fish, shellfish, peanut, tree nut, fruit, veggies, legumes, grains, other (1-13):

Foods avoided due to IgE allergy: egg, milk, wheat, soya, fish, shellfish, peanut, tree nut, fruit, veggies, legumes, grains, other (1-13)

% on elimination diet for EoE

% on elimination diet to multiple foods

number of foods being avoided (& %)

Number/% with asthma

Number/% had eczema previously

Number/% with eczema

Number/% with rhintis

% with family atopy backgrounds:

age range at start of study

median age at start of study

age range of start of EoE symptoms

median age at start of EoE symptoms

age range for diagnosis of EoE

median age at diagnosis of EoE

median delay time for EoE diagnosis

age range for start of feeding difficulty symptoms

median age of start of feeding difficulty symptoms

age range at diagnosis of feeding difficulty

median age at diagnosis of feeding difficulty

median delay time for feeding difficulty diagnosis:

% with previous anaphylaxis to foods

% with AAI for food allergy

% in remission of food allergy:

Feeding difficulties

number of different feeding difficulties assessed in study

terminology/names of feeding difficulties assessed

definition of feeding difficulty/ies used

criteria used for diagnosis of feeding difficulties: Wolfson, Chatoor, BPFAS, CEBQ, others?

Means of confirming feeding difficulty: parental report, healthcare questionnaire, medical records, specialist-assessed eating study

% with feeding difficulties reported by parents only

% of reported difficulties that were confirmed with physician diagnosis

% prevalence of feeding difficulties (with CI)

%/number with 1 specified feeding difficulty phenotype

%/number with >1 specified feeding difficulty phenotype

Specific phenotype of feeding difficulty (if defined)

%prevalence of selective eating

%prevalence of prevalence of eating too little/no appetite

%prevalence of fear of food

% prevalence of food refusal %prevalence of aversive eating

%prevalence of food neophobia

%prevalence of maladaptive feeding %prevalence of fussy eating

%prevalence of picky eating

% prevalence of avoidant eating

%prevalence of ARFID

%prevalence of paediatric feeding disorder

%prevalence with unspecified/generic feeding difficulty/problem

EoE specific information

% with extra-GI symptoms e.g. joint mobility

%/number with abdominal pain

%/number with constipation

%/number with diarrhoea

%/number with dysphagia

%/number with food impaction

%/number with reflux

%/number with vomiting

%/number with rumination

%/number with 'adaptive feeding'

%/number being 'slow eater'

%/number with cutting food into small pieces

%/number with drinking excessive water

%/number with texture hypersensitivity

%/number with excessive chewing / 'pouching'

%/number with respiratory complications

%/number with aspiration

%/number with recurrent chest infections

%/number with wheezing suspectedly due to reflux/swallowing issues

%/number taking medication for their EoE

%/number with ENT complications (recurrent ear infections, hoarseness )

Comorbidities

%/number with other comorbidities potentially impacting on feeding/eating

%/number with ASD

%/number with neuro-disability

%/number with an eating disorder (anorexia nervosa, bulimia, binge eating)

Treatment

% receiving treatment for feeding difficulties

Treatment received for feeding difficulties – dietician support, counselling, CBT, family treatment (1-4)

Median time to treat feeding difficulty after diagnosis

Median age when treatment for feeding difficulties started

% in remission of feeding difficulty

Median duration of feeding difficulty symptoms

Impact of feeding difficulties

any height / weight parameters reported (yes / no)

weight-for-age

height-for-age

weight-for-height

BMI z-scores/SD

growth impact (faltering/malnutrition/FTT/weight loss)

obesity

QoL

hours of feeding

%/number of patients with mental health issues

mental health issues suffered by patient: depression, anxiety, suicidal ideation, social isolation, PTSD (1-5)

%/number of parents with mental health issues

mental health issues of parents/family: depression, anxiety, suicidal ideation, social isolation, PTSD (1-5)

school absenteeism

parental/carer work absenteeism

other effects of note

Study conclusion

Concluding message(s):

**REFERENCES (Supplemmentary Material)**

1. Barfjani, S. H., et al. (2016). "Esophagitis in infants and toddlers with feeding difficulties: Prevalence and associated clinical characteristics." Journal of Pediatric Gastroenterology and Nutrition 63(Supplement 2): S18-S19.
2. Beser, O. F., et al. (2011). "Evaluation of 20 cases who have eosinophilic esophagitis." Acta Paediatrica, International Journal of Paediatrics 46(3): 42.
3. Guarnere, S., et al. (2020). "Speech-language pathologists promote management of children with eosinophilic esophagitis." American Journal of Gastroenterology 115(SUPPL): S609
4. Hilow, E., et al. (2019). "Natural history of eosinophilic esophagitis in children: A retrospective chart review from a tertiary care center." Journal of Pediatric Gastroenterology and Nutrition. Conference: North American Society for Pediatric Gastroenterology, Hepatology and Nutrition Annual Meeting, NASPGHAN 69(Supplement 2).
5. Hoofien, A., et al. (2019). "Pediatric Eosinophilic Esophagitis: Results of the European Retrospective Pediatric Eosinophilic Esophagitis Registry (RetroPEER)." Journal of Pediatric Gastroenterology and Nutrition 68(4): 552-558.
6. Levin, M. and C. Motala (2011). "Eosinophilic esophagitis in Cape Town, South Africa." Clinical and Translational Allergy. Conference: Food Allergy and Anaphylaxis Meeting 1(SUPPL. 1).
7. Menzies, J., et al. (2017). "Prevalence of malnutrition and feeding difficulties in children with esophageal atresia." Journal of Pediatric Gastroenterology and Nutrition 64(4): e100-e105.
8. Pham, A., et al. (2019). "Feed-easy: Feeding disorders in children with esophageal atresia study." Diseases of the Esophagus 32(Supplement 1): 8-9.
9. Taylor, J. and E. Volonaki (2019). "Incidence of eosinophilic oesophagitis in a UK paediatric population over a 10-year period." Journal of Pediatric Gastroenterology and Nutrition 68(Supplement 1): 313.
10. Brytek-Matera, A., et al. (2022). "Symptoms of Avoidant/Restrictive Food Intake Disorder among 2-10-Year-Old Children: The Significance of Maternal Feeding Style and Maternal Eating Disorders." Nutrients 14(21): 13.
11. Chudoba, A. (2022). "Eosinophilic gastrointestinal diseases in childhood." Pediatria I Medycyna Rodzinna-Paediatrics and Family Medicine 18(2): 119-124.
12. Ciciulla, D., et al. (2023). "Systematic Review of the Incidence and/or Prevalence of Eating Disorders in Individuals With Food Allergies." The Journal of Allergy & Clinical Immunology in Practice 11(7): 2196-2207.e2113.
13. Cifra, N. and J. Lomas (2022). "158. Case Series: Eosinophilic Esophagitis Presenting as an Eating or Feeding Disorder." Journal of Adolescent Health 70(4 Supplement): S83.
14. Cifra, N. and J. M. Lomas (2022). "Differentiating Eosinophilic Esophagitis and Eating/Feeding Disorders." Pediatrics 149(4): 01.
15. Ciurez, B. T., et al. (2023). "Risk Factors Related to Eating Disorders in a Romanian Children Population." Nutrients 15(13): 10.
16. Dumont, E., et al. (2022). "Feeding/Eating Problems in Children Who Refrained From Treatment in the Past: Who Did (Not) Recover?" Frontiers in Pediatrics 10 (no pagination).
17. Hollaender, M., et al. (2022). "The incidence of eosinophilic oesophagitis in 2007-2017 among children in North Denmark Region is lower than expected." BMC Pediatrics 22(1) (no pagination).
18. Keles, M. N., et al. (2023). "Oropharyngeal Dysphagia in Children with Eosinophilic Esophagitis." Dysphagia 38(1): 474-482.
19. Koenigsberg, R., et al. (2022). "Body mass index in relation to presenting symptoms and age upon diagnosis of Eosinophilic Esophagitis." Journal of Allergy and Clinical Immunology 149(2 Supplement): AB205.
20. Lucendo, A. J., et al. (2022). "EoE CONNECT, the European Registry of Clinical, Environmental, and Genetic Determinants in Eosinophilic Esophagitis: rationale, design, and study protocol of a large-scale epidemiological study in Europe." Therapeutic Advances in Gastroenterology 15(no pagination).
21. Monteiro, A., et al. (2023). "How maternal personality traits can affect children's neophobia to milk and egg food allergies?" Allergy: European Journal of Allergy and Clinical Immunology 78(Supplement 111): 484-485.
22. Moroco, A. E. and N. L. Aaronson (2022). "Pediatric Dysphagia." Pediatric Clinics of North America 69(2): 349-361.
23. Muir, A. B., et al. (2023). "It takes a village to manage eosinophilic esophagitis." Annals of Allergy, Asthma and Immunology 130(1): 13-14.
24. Patrawala, M. M., et al. (2022). "Avoidant-restrictive food intake disorder (ARFID): A treatable complication of food allergy." Journal of Allergy and Clinical Immunology: In Practice 10(1): 326-328.e322.
25. Philpott, H., et al. (2022). "Patient demographics and management of eosinophilic esophagitis in Australasian children: Racial differences and regional patterns of care vary across all capital cities in a decade of data." Journal of Gastroenterology and Hepatology 37(Supplement 1): 211.
26. Rodrigues, V. C. C., et al. (2022). "Feeding difficulties in children fed a cows' milk elimination diet." British Journal of Nutrition 128(6): 1190-1199.
27. Sitarik, A. R., et al. (2021). "Infant Feeding Practices and Subsequent Dietary Patterns of School-Aged Children in a US Birth Cohort." Journal of the Academy of Nutrition and Dietetics 121(6): 1064-1079.
28. Votto, M., et al. (2022). "Eosinophilic esophagitis an update in children." Acta Bio-Medica de l Ateneo Parmense 93(S3): e2022034.
29. Yong, C., et al. (2023). "Parental food neophobia, feeding practices, and preschooler's food neophobia: A cross-sectional study in China." Appetite 185 (no pagination).
30. The Effective Public Health Practice Project (EPHPP). Quality assessment tool for quantitative studies. Accessed June 2020. http://www. ephpp. ca/ tools. Html
